# Giant cell arteritis–associated temporomandibular joint arthritis confirmed by imaging

**DOI:** 10.1093/rap/rkag029

**Published:** 2026-03-09

**Authors:** Miyu Wakatsuki, Hiroyuki Yamashita, Ami Isoda, Takuya Harada, Hiroshi Kaneko

**Affiliations:** Division of Rheumatic Diseases, National Center for Global Health and Medicine, Shinjuku, Tokyo, Japan; Division of Rheumatic Diseases, National Center for Global Health and Medicine, Shinjuku, Tokyo, Japan; Division of Rheumatic Diseases, National Center for Global Health and Medicine, Shinjuku, Tokyo, Japan; Division of Rheumatic Diseases, National Center for Global Health and Medicine, Shinjuku, Tokyo, Japan; Division of Rheumatic Diseases, National Center for Global Health and Medicine, Shinjuku, Tokyo, Japan

Key messages
• Temporomandibular joint arthritis in giant cell arteritis can initially present as trismus.

Dear Editor, Giant cell arteritis (GCA) is a large-vessel vasculitis that typically presents with headache and jaw claudication. As GCA can result in blindness, early diagnosis and prompt initiation of treatment are crucial [1]. Patients with GCA may also present with reduced jaw opening (i.e. trismus) [2], but trismus is rarely recognized as a symptom of GCA and is often misattributed to other causes, resulting in diagnostic delay [3]. There is long-standing uncertainty regarding the mechanism of underlying trismus in GCA. This case report describes a patient with GCA initially presenting as trismus, while both MRI and FDG-PET/CT demonstrated enhancement of the temporomandibular joint (TMJ), indicating TMJ arthritis.

A 78-year-old woman presented with a 1-month history of trismus, which was initially diagnosed with TMJ arthritis and treated with antibiotics by dental and otolaryngology specialists, without resolution of symptoms. Afterwards, the patient developed low-grade fever and scalp tenderness, as well as diplopia for 1 week, prompting admission to our hospital for further evaluation. Upon admission, there was mild dilatation and tenderness of the temporal artery, left abducens nerve palsy, pain in both TMJ regions, and trismus (maximum unassisted mouth opening: 28 mm). Laboratory tests showed elevated C-reactive protein (CRP) (10.05 mg/dl) and ESR (>120 mm/h). Temporal artery ultrasound revealed a halo sign. Contrast-enhanced T1-weighted MRI demonstrated high signal intensity in the superficial temporal artery with a corresponding high signal on diffusion-weighted imaging, suggestive of GCA. Temporal artery biopsy revealed marked inflammatory cell infiltration of the vessel wall, severe wall thickening with near luminal occlusion, disruption of the internal elastic lamina, and occasional multinucleated giant cells, leading to the diagnosis of GCA (Fig. 1A–C). Notably, contrast-enhanced T1-weighted MRI revealed high signal intensity in the posterior wall of the TMJ (Fig. 1D), and the TMJ also had increased fluorodeoxyglucose uptake on FDG-PET/CT (Fig. 1E). These findings implicate TMJ arthritis as the cause of trismus. Other potential causes of TMJ inflammation, including inflammatory arthritis (e.g. rheumatoid arthritis and infectious arthritis), were considered. However, the absence of arthritis in other joints, negative rheumatoid serology, imaging findings, and the clinical course made these diagnoses unlikely. Treatment was started with intravenous methylprednisolone pulse therapy (250 mg daily for 3 days), followed by oral prednisolone (40 mg). Afterwards, there was prompt resolution of headache, TMJ arthritis, and trismus along with decreased CRP levels. The patient was discharged on day 20. Follow-up FDG-PET/CT at 3 months was performed to assess overall disease activity of GCA and to evaluate for newly developed vascular involvement or alternative diagnoses. The scan revealed complete resolution of fluorodeoxyglucose uptake in the TMJ (Fig. 1F), with no abnormal FDG uptake elsewhere.

**Figure 1:**
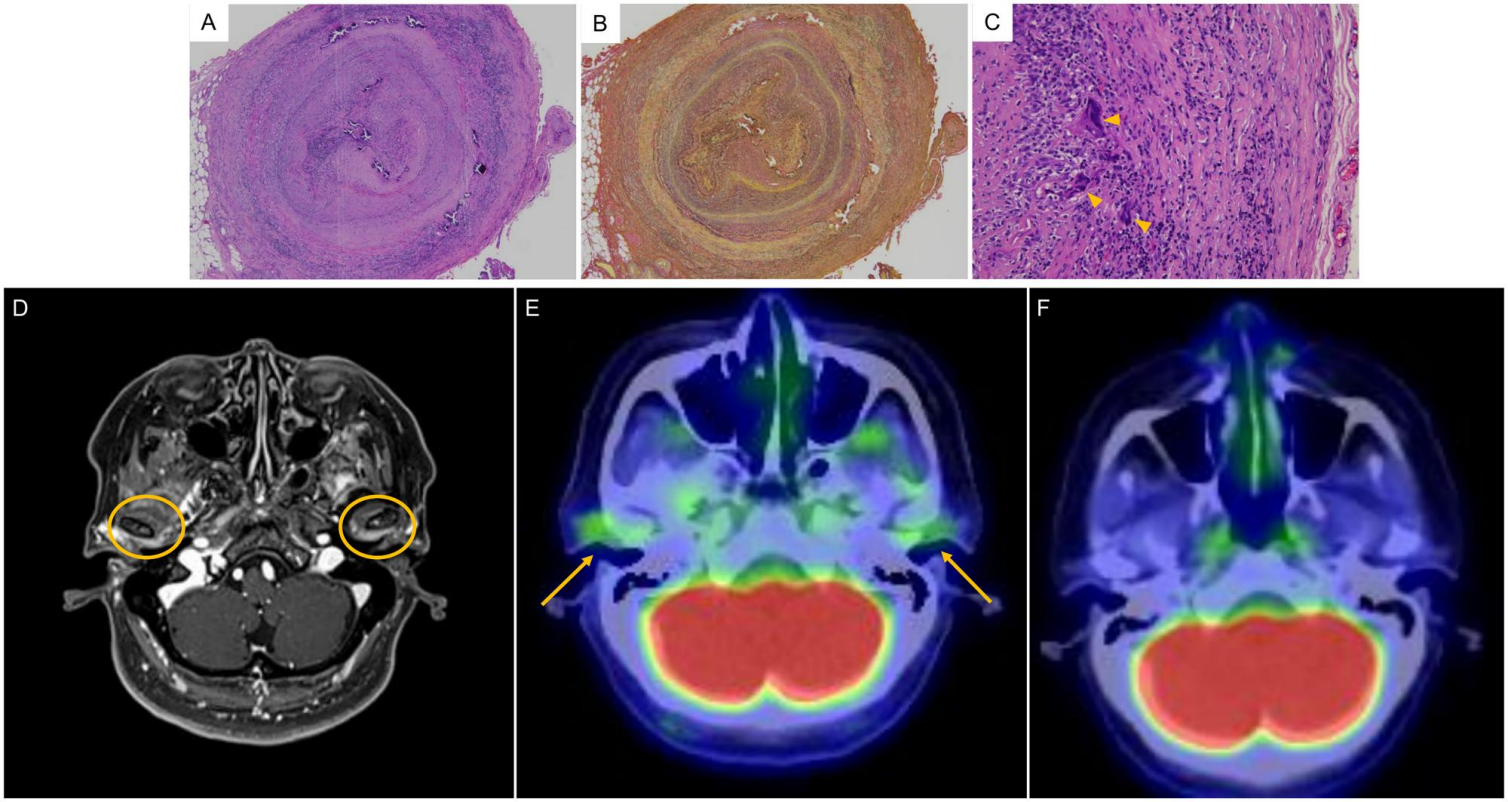
Histological findings and imaging. Temporal artery biopsy revealed marked inflammatory cell infiltration and severe thickening of the vessel wall with near luminal occlusion (A, H&E staining; original magnification ×12.5), disruption of the internal elastic lamina (B, elastica van Gieson staining; original magnification ×12.5), and multinucleated giant cells (C, H&E staining; original magnification ×200). Contrast-enhanced T1-weighted MRI revealed high signal intensity in the posterior wall of the temporomandibular joint (D). FDG-PET/CT demonstrated increased fluorodeoxyglucose uptake in the temporomandibular joint, supporting the diagnosis of temporomandibular joint arthritis (E). Three months after starting therapy, repeat FDG-PET/CT revealed complete resolution of FDG uptake in the same region (F)

Unlike jaw claudication, trismus is a rare symptom of GCA, observed in only 6.8% of cases [2]. Trismus has previously been associated with the ocular manifestations of GCA, but another study found no such relationship [4]. Diplopia, which arises from ischaemia of the branches of the ophthalmic artery, occurs in 5–10% of patients with GCA [5] and serves as a warning sign preceding blindness [6]. In this case, the patient developed diplopia after trismus due to abducens nerve palsy. Previous reports of GCA presenting with trismus noted a diagnostic delay of 1–2 months, with symptom improvement after prednisolone therapy [7, 8].

Trismus in GCA is often overlooked, and its underlying mechanism remains unclear. Some hypotheses include ischaemia of the maxillary artery supplying the masseter muscle, as well as involvement of the trigeminal and facial nerves [7, 8]. To our knowledge, this is the first report of GCA presenting with trismus and evidence of TMJ arthritis on both FDG-PET/CT and MRI. The TMJ is supplied by branches of the external carotid artery, such as the superficial temporal and maxillary arteries [9], which are often involved in giant cell arteritis. In the present case, imaging confirmed that inflammatory changes were localized to the TMJ. These findings support the possibility that localized inflammatory TMJ arthritis associated with GCA may contribute to trismus. In conclusion, trismus should be recognized as a possible clinical manifestation of GCA, wherein localized TMJ arthritis is a potential underlying mechanism.

## Data Availability

Data are available from the corresponding author upon request.
